# IL-21 modulates memory and exhaustion phenotype of T-cells in a fatty acid oxidation-dependent manner

**DOI:** 10.18632/oncotarget.24442

**Published:** 2018-02-07

**Authors:** Romy Loschinski, Martin Böttcher, Andrej Stoll, Heiko Bruns, Andreas Mackensen, Dimitrios Mougiakakos

**Affiliations:** ^1^ Department of Internal Medicine 5, Hematology and Oncology, University of Erlangen-Nuremberg, Erlangen, Germany

**Keywords:** Interleukin-21, Interleukin-2, T-cell metabolism, memory T-cell, PD-1

## Abstract

T-cell-based therapies represent a promising strategy for cancer treatment. In this context, cytokines are discussed as a bona fide instrument for fine-tuning T- cell biology. One promising candidate is the pleiotropic interleukin-21 (IL-21) with only little being known regarding its direct effects on human T-cells. Thus, we sought out to characterize the impact of IL-21 on T-cell metabolism, fitness, and differentiation. Culturing T-cells in presence of IL-21 elicited a metabolic skewing away from aerobic glycolysis towards fatty acid oxidation (FAO). These changes of the metabolic framework were paralleled by increased mitochondrial fitness and biogenesis. However, oxidative stress levels were not increased but rather decreased. Furthermore, elevated FAO and mitochondrial biomass together with enhanced antioxidative properties are linked to formation of longer lasting memory responses and less PD-1 expression. We similarly observed an IL-21-triggered induction of central memory-like T-cells and reduced levels of PD-1 on the cell surface. Taken together, IL-21 shifts T-cells towards an immunometabolic phenotype that has been associated with increased survivability and enhanced anti-tumor efficacy. In addition, our data reveals a novel interconnection between fatty acid metabolism and immune function regulated by IL 21.

## INTRODUCTION

To date, adoptive T-cell transfer has emerged as an important pillar for immune-based therapies in malignant diseases. In fact, *ex vivo* modified and expanded T-cells have shown promising activity in cancer patients that are otherwise resistant to conventional therapy [1]. However, several hurdles still exist that need to be overcome. Tumor cells employ a broad variety of strategies for evading intrinsic immunity and immune-based therapies including lack of antigen presentation, induction of immune regulatory cell subsets, and metabolic interferences [2]. Notably, several current studies emphasize the importance of the tumor-associated metabolic re-modelling of the tumor microenvironment. It ranges from metabolic competition over critical nutrients such as glucose and tryptophan to the abundant production of toxic metabolic byproducts including reactive oxygen species (ROS) [3–5]. Therefore, it is necessary to develop strategies not only for improving the targeting functions of the transferred T-cells but also their survivability and metabolic robustness.

To this end, cytokines have been exploited based on their manifold T-cell promoting functions. Especially the common gamma chain cytokines play a pivotal role in T-cell differentiation, expansion, and functionality. Thus, interleukin-2 (IL-2) is already approved as an *in vivo* T-cell modulator for the treatment of patients with metastatic melanoma and renal cell carcinoma [6–10]. However, it is being put under scrutiny due to its (mainly *in vivo*) tolerogenic effects by promoting generation and peripheral expansion of Tregs [11]. Interleukin-15 (IL-15), also member of this cytokine family, has been widely investigated regarding its more favorable properties as compared to IL-2. Several studies found that IL-15 promotes longer lasting memory T-cell responses, which are associated with an increased antioxidative capacity and therefore better survivability within a hostile tumor microenvironment [12, 13]. Moreover, Kesarwani and colleagues reported that equipping T-cells with an increased antioxidative capacity is beneficial for *in vivo* tumor control [13]. In addition, recent observations suggest that chimeric antigen receptor-carrying T-cells benefit from an enhanced expression of antioxidants [14]. Redox status, differentiation, function, and consequently the anti-tumor activity are determined by the metabolic status of the T-cells [15]. Isolating T-cells based on metabolic features for cellular therapies could represent an elegant approach [16]. In general, effector T-cells immediately switch towards aerobic glycolysis upon activation. Contrariwise, long-lasting memory-like T-cells rely preferentially on mitochondrial oxidative phosphorylation (OXPHOS) and fatty acid oxidation (FAO) for meeting their energetic demands [17, 18].

IL-21, another member of the common gamma chain cytokine family, has also been shown to exert beneficial effects on T-cell function. In this context, an increasing number of studies highlight its role in driving memory formation in mice [19, 20]. In addition, suppressive effects on development and homeostasis of regulatory T-cells (Tregs), which regularly accumulate in cancer patients, were documented in *in vitro* and *in vivo* experiments [21, 22]. However, the underlying mechanisms and in particular its metabolic effects are not fully understood yet. Therefore, we focused on the potential IL-21 mediated changes of the T-cells’ metabolism in a direct head-to-head comparison with the clinically established IL-2.

Treating T-cells with IL-21 led to a metabolic skewing away from aerobic glycolysis towards FAO. This metabolic reprogramming was accompanied by an increased mitochondrial biogenesis and a superior mitochondrial fitness. Interestingly, cellular antioxidants were elevated explaining the overall lower levels of intracellular ROS. In accordance to previous observations we found the aforementioned metabolic alterations to be linked with a preferential induction of central memory-like T-cells and reduced exhaustion/senescence. Key IL-21-related findings were also reproduced in T-cells from patients with chronic lymphocytic leukemia (CLL). With CLL being the most common leukemia in adults featuring alterations, such as oxidative stress and senescent T-cells, these effects could be advantageous for an anti-leukemic T-cell function [4, 23]. Taken together, we herewith describe for the first time several beneficial immune metabolic effects in T-cells, which are elicited by IL-21. Our results constitute a solid foundation for further exploiting those IL-21-triggered effects especially in view of T-cell-based therapeutic approaches.

## RESULTS

### IL-21 skews T-cell metabolism towards FAO

**Figure 1:**
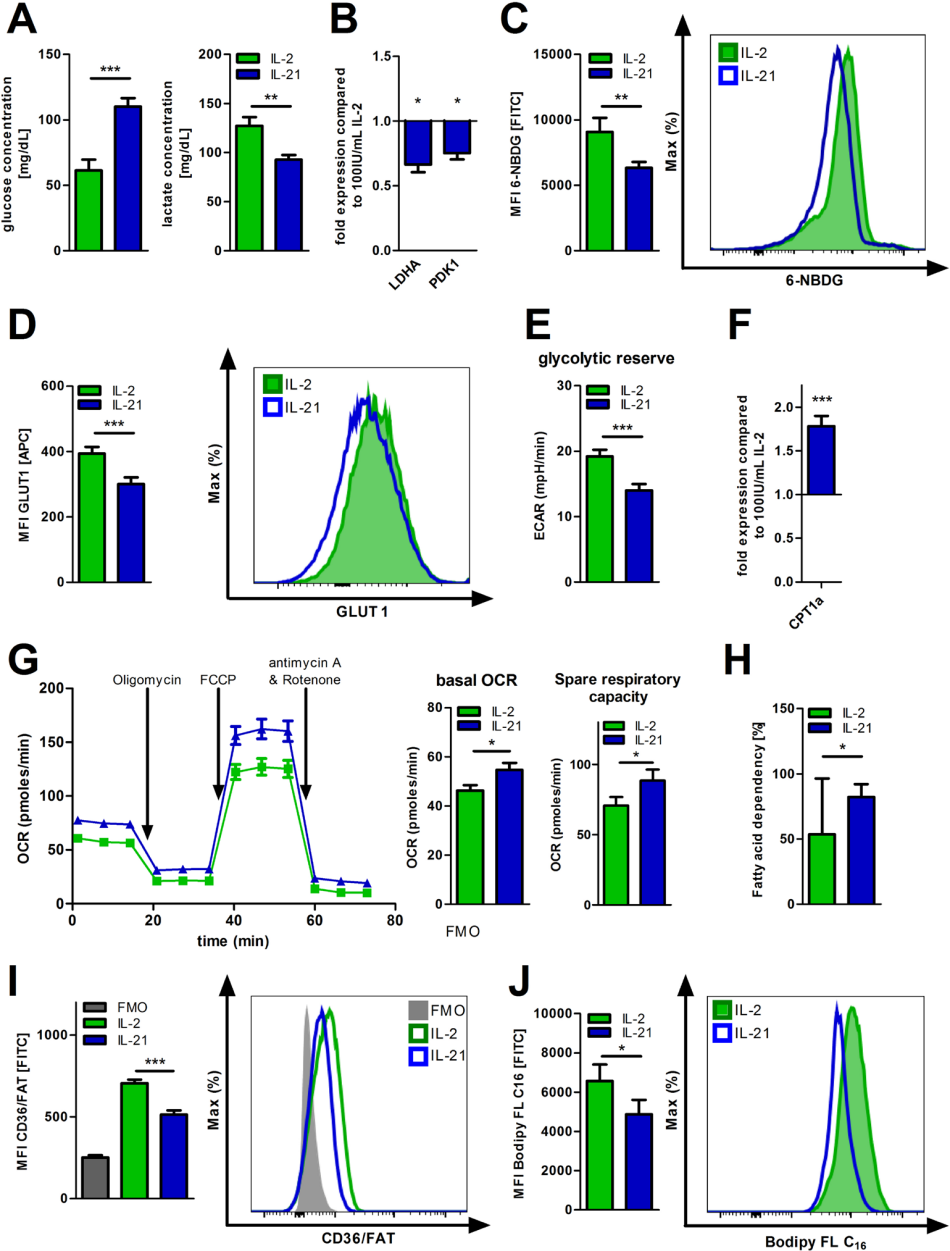
Metabolic reprogramming of IL-21 treated T-cells (**A**–**J**) T-cell culture was performed over 5 days with IL-2 or IL-21 in the presence of activation/expansion beads. (A) Glucose and lactic acid concentrations were determined in the supernatants of IL-21 treated, activated T-cells compared to IL-2 treatment with HITADO Super GL compact (*n* = 6). (B) After harvesting, lysis and RNA isolation of T-cells, mRNA expression levels of LDHA and PDK1 were analyzed by qPCR (*n* = 6). (C) Glucose uptake was semi-quantified by flow cytometry using 6-NBDG (*n* = 6). Left: Summary of MFIs from 6 different donors. Right: representative histogram of one donor. (D) The surface expression of GLUT1 was detected by flow cytometric staining. Left: Summary of MFIs from 6 different donors. Right: representative histogram of one donor. (E) ECAR was measured performing the Glycolysis Stress Test under basal conditions and after inhibitor injections as indicated. Afterwards, glycolytic reserve was calculated. (*n* = 3). (F) The relative CPT1a mRNA expression was assessed from T-cell lysates obtained from IL-21 cell cultures in comparison to IL-2 (*n* = 6). (**G**) OCR was assessed performing the Mito Stress Test under basal conditions and after inhibitor injections as indicated. Afterwards, basal respiration and SRC were calculated as specified by Seahorse Bioscience (*n* = 3). (H) The dependency of OXPHOS on fatty acid oxidation was measured and calculated using the Seahorse Mito Fuel Flex Test (*n* = 3). Error bars indicate the standard deviation. (I) Surface expression of CD36/FAT was analyzed by flow cytometry (*n* = 6). Left: Summary of MFIs from 6 different donors. Right: representative histogram of one donor. (J) For studying uptake of long chain fatty acids, cells were incubated with a fluorescently labeled C_16_ fatty acid (Bodipy FL C_16_) and analyzed by flow cytometry (*n* = 9). Left: Summary of MFIs from 9 different donors. Right: representative histogram of one donor. MFI is defined as median fluorescence intensity. Unless stated otherwise, error bars indicate standard error means. *P* value: ^*^P < .05; ^**^P < .01; ^***^P < .001

The common gamma chain cytokines IL-2, IL-7, and IL-15 have been found to impact T-cell metabolism. Therefore, we investigated whether expanding T-cells in presence of IL-21 (as compared to IL-2) changes their metabolic phenotype. Supernatants from IL-21 treated T-cells showed less glucose consumption and as anticipated less lactic acid release (Figure 1A, Supplementary Figure 1A). In accordance with this data, expression of lactate dehydrogenase (LDHA), a key enzyme of aerobic glycolysis, was also found reduced (Figure 1B). In fact, expression of pyruvate dehydrogenase kinase (PDK1), which inhibits the conversion of pyruvate into acetyl-CoA for fueling OXPHOS, is also downregulated (Figure 1B). Furthermore, both glucose uptake on single T-cell level and surface density of the key glucose transporter 1 (GLUT1) were negatively impacted by IL-21 (Figure 1C–1D). Taken together, IL-21 mediated effects of various components of glycolysis yielded a significantly overall reduced glycolytic potency, which is further revealed by our dynamic flux analyses measuring the extracellular acidification rate (ECAR), an indicator for aerobic glycolysis (Figure 1E). Metabolic competence is of fundamental importance for proper T-cell function. Despite the apparent changes of their glucose metabolism, T-cells treated with IL-21 displayed a similar responsiveness as their IL-2 treated counterparts towards activating stimuli (Supplementary Figure 1B, Supplementary Figure 1C). When culturing T-cells with IL-2 or IL-21 in media containing different glucose levels we did not notice reduced proliferation of the IL-21 population (under rather glucose-deprived conditions). In fact, IL-21 expanded CD8^+^ T-cells proliferated at significantly higher rates regardless the glucose concentration (Supplementary Figure 1D). However, we observed a significantly higher expression of the carnitine palmitoyltransferase 1a (CPT1a), the rate-limiting enzyme for β-oxidation of fatty acids (FAO) that transfers cytosolic fatty acids into mitochondria [24] (Figure 1F, Supplementary Figure 1E). Next, we examined the effect of uncoupling respiration from energy producing OXPHOS using FCCP. T-cells expanded in presence of IL-21 displayed a larger mitochondrial spare respiratory capacity (SRC) as indicated by the difference between the maximal OCR (upon FCCP injection) and basal OCR (Figure 1G). In addition, dependency on fatty acids for fueling mitochondrial oxidation was enhanced as opposed to glucose and glutamine when evaluating OCR changes upon blocking FAO, glycolysis, and glutaminolysis respectively (Figure 1H, Supplementary Figure 1F). To our surprise, surface expression of fatty acid transporter CD36/FAT was diminished (Figure 1I). In accordance, the uptake of fluorescently labeled palmitate (Bodipy FL C_16_/long chain fatty acids) was significantly decreased (Figure 1J) pointing towards a preferential utilization of intrinsic (and not extrinsic) fatty acids. To further validate this finding we designed an according metabolic flux-based assay. BSA-conjugated palmitate was added to the T-cell cultures just before starting OCR measurements. While detecting OCR, SSO, an inhibitor of CD36 and the uptake of fatty acids, was injected to the cells. Next, cells were treated with Etomoxir, which blocks transport of both intrinsic and extrinsic fatty acids into mitochondria for being oxidized. In fact, only CPT1a inhibition had an impact on OCR (i.e. FAO) further corroborating the notion that IL-21 treated T-cells preferentially utilize intrinsic fatty acids for FAO (Supplementary Figure 1G).

### IL-21 promotes mitochondrial biogenesis and mitochondrial fitness

**Figure 2:**
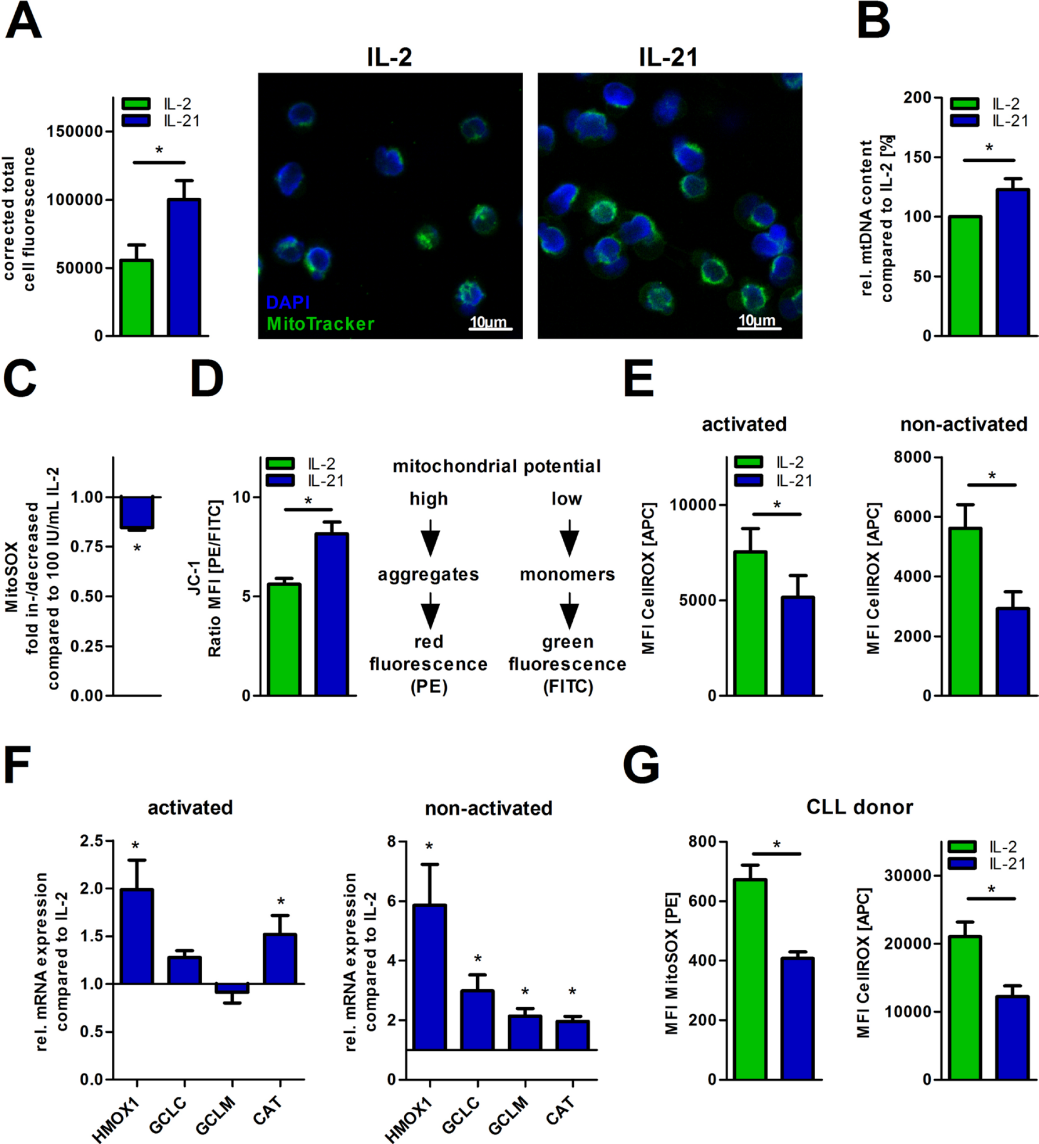
IL-21 increases mitochondrial biogenesis and fitness (**A**–**F**) T-cell cultivation was performed over 5 days with IL-2 or IL-21 in the presence of activation/expansion beads. (A) Mitochondrial mass was visualized using MitoTracker™ in cytokine-treated T-cells by fluorescence microscopy as shown for one representative donor. Samples were also counterstained with the nuclear dye DAPI. Quantification of MitoTracker™ fluorescence was done with ImageJ for 5 single cells per condition. (B) The content of mtDNA was determined as the mitochondrial DNA copy number relative to nuclear DNA using qPCR analysis (*n* = 6). (C) The mitochondrial superoxide production was semiquantified using MitoSOX™ probe with flow cytometry (*n* = 6). (D) The mitochondrial membrane potential (∆ΨM) was semiquantified by flow cytometry using the potentiometric dye JC-1 (*n* = 6). In mitochondria with a high membrane potential JC-1 forms aggregates which exhibit a fluorescence emission of higher wavelength (approx. 590 nm). In mitochondria with a low membrane potential JC-1 aggregates separate which feature a shift of the emission to lower wavelength (approx. 530 nm). Hereby, the ratio of both red and green fluorescence allows comparing the membrane potential between the two cell culture conditions. (E) Total intracellular ROS content was detected in IL-21 treated T-cells compared to IL-2 under both non-activating and activating conditions by flow cytometry (*n* = 6). (F) The relative gene expression of key cellular antioxidants (catalase, CAT; heme-oxygenase-1, HMOX1; glutamate cysteine ligase catalytic subunit, GCLC; glutamate cysteine ligase modifier subunit, GCLM) is shown for IL-21 treated T-cells in relation to IL-2 treated counterparts as quantified by qPCR (*n* = 6). (**G**) CLL PBMCs with less than 70% CD5^+^ B-cells were cultured for 5 days in the presence of IL-2 or IL-21 with anti-CD2/CD3/CD28 beads. Total cells were incubated with MitoSOX™ for mitochondrial superoxide production and CellROX™ for total intracellular ROS level. For analysis of T-cells, cells were gated according to their CD3 expression. MFI is defined as median fluorescence intensity. Error bars indicate the standard error means. *P* value: ^*^P < .05; ^**^P < .01; ^***^P < .001

Decreased aerobic glycolysis during generation of CD8^+^ memory T-cells using IL-15 has been shown to be accompanied by altered mitochondrial biology as compared to IL-2 primed effector T-cells [17]. Hereby, increased FAO and SRC have been previously linked to an enhanced mitochondrial biogenesis [17]. In fact, we observed increased mitochondrial biomass in IL-21 treated cells as assessed by fluorescence microscopy (Figure 2A). Cell size did not change significantly (Supplementary Figure 2A). In accordance with this finding, the ratio of nuclear (n) to mitochondrial (mt) DNA was significantly shifted towards the latter when compared to IL-2 (Figure 2B). Lower mitochondrial production of reactive oxygen species (ROS) and the ability to maintain the mitochondrial potential represent two well-established surrogates for mitochondrial fitness. Mitochondrial ROS production decreased while mitochondrial potential increased in presence of IL-21 (Figure 2C–2D). Lower mitochondrial ROS levels upon IL-21 treatment, as shown before, were paralleled by reduced total intracellular ROS levels (Figure 2E). Remarkably, decreased total oxidative stress was also observed in T-cells, which received no additional TCR stimulus (termed as “non-activated”, Figure 2E). These redox effects could also be attributed to an upregulation of key antioxidants such as heme-oxygenase 1 (HO-1), glutamate-cysteine ligase catalytic and regulatory subunit (GCLC/GCLM), and catalase (CAT) (Figure 2F). Overall, IL-21 treated T-cells display a higher resilience towards ROS-induced cell death (Supplementary Figure 2B). Additionally, we observed an increased expression of the anti-apoptotic protein Bcl-2, but not of Mcl1 and of Bcl-xL (Supplementary Figure 2C). Furthermore, we were interested if we could see similar effects concerning redox capacities in CLL patient-derived T-cells that were already challenged by their ROS-rich environment. Although IL-21 serum levels are not found reduced in CLL patients (as compared to healthy controls) (Supplementary Figure 2D), recent studies indicate that promoting the IL-21 production might have a beneficial impact in terms of CLL-cell-directed cytotoxicity [25]. Indeed, we observed decreased mitochondrial and total cellular ROS-levels in T-cells from CLL PBMCs cultured with IL-21 (Figure 2G).

### IL-21 reduces markers of T-cell exhaustion and senescence

**Figure 3:**
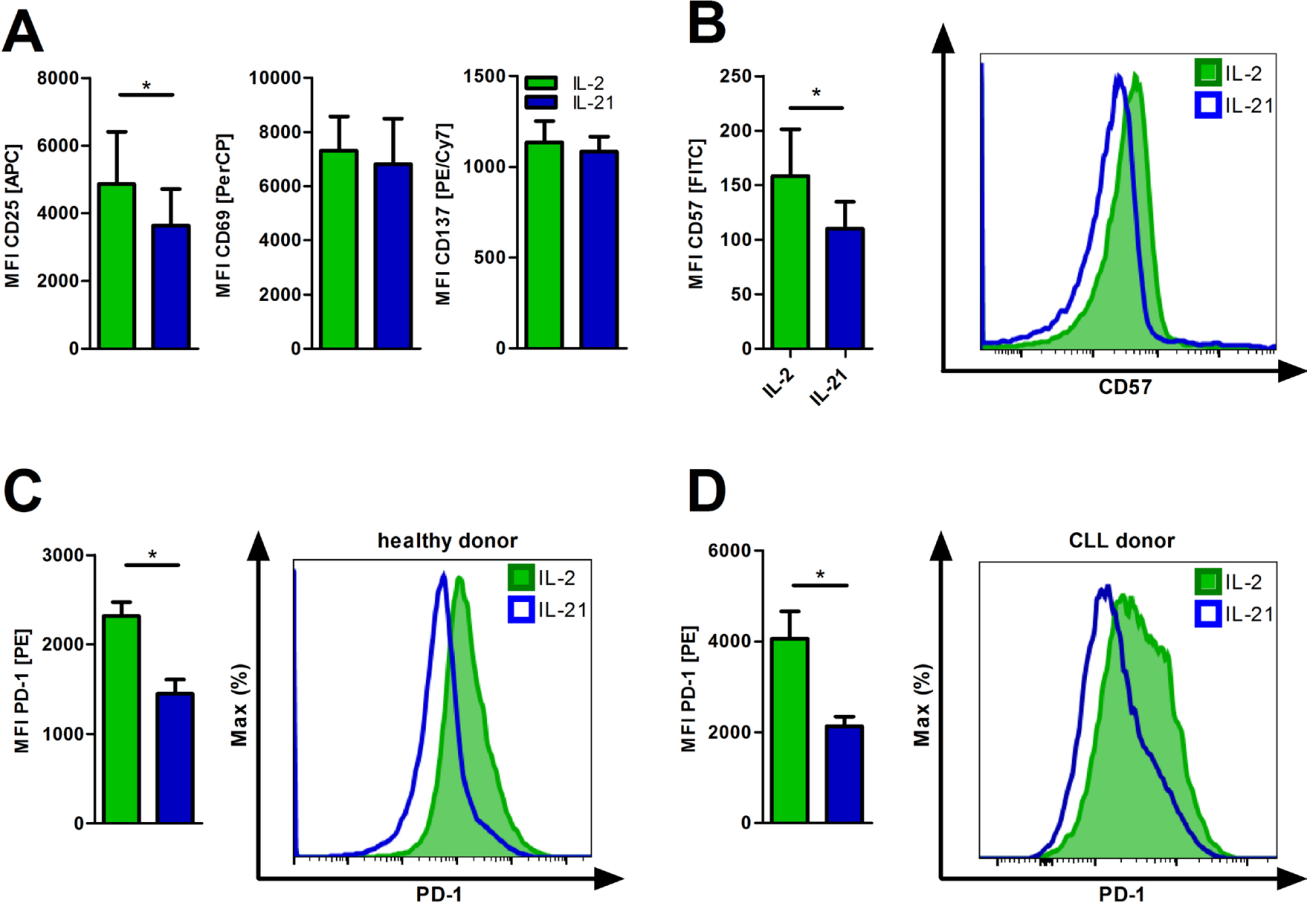
IL-21 diminishes T-cell exhaustion and senescence (**A**) T-cells cultured in the presence of IL-2 or IL-21 were activated for 24 hours and subsequently analyzed for the expression of the activation markers CD25, CD69 and CD137 (*n* = 6). (**B**–**C**) T-cells were cultured for 5 days in the presence of IL-2 or IL-21 with anti-CD2/CD3/CD28 activation/expansion beads. Afterwards, cells were harvested and examined for surface expression of CD57 (B) and PD1 (C) by flow cytometry (*n* = 6). (D) CLL PBMCs with less than 70% CD5^+^ B-cells were cultured for 5 days in the presence of IL-2 or IL-21 with anti-CD2/CD3/CD28 beads. T-cells were distinguished by CD3 expression and analyzed for surface PD-1 levels (*n* = 6). MFI is defined as median fluorescence intensity. Error bars indicate the standard error mean. *P* value: ^*^P < .05.

As a member of the common gamma chain cytokine family, IL-21 is known to play a pleiotropic role in immunoregulation and -modulation [26]. Therefore, we studied whether IL-21 influences the activation status of T-cells as compared to the commonly used growth factor IL-2. Activation markers were assessed after 24 hours of incubation via flow cytometry. Expression of CD69 and CD137 was similar while CD25 was found downregulated (Figure 3A) with the proliferative capacity not being affected (Supplementary Figure 1B).

T-cell senescence and (pseudo-)exhaustion are regularly described in tumor patients including CLL [27] and represent an important immune escape mechanism. They are caused by chronic activation and/or inhibitory signals. We cultured T-cells under conditions of persistent activation for five days by adding stimulatory anti-CD2/CD3/CD28 beads +IL-2 or +IL-21 and evaluated expression of senescence and exhaustion markers. Incubation with IL-21 significantly decreased surface expression of CD57, a marker for replicative senescence [28], and of PD-1 (Figure 3B–3C). Interestingly, adding IL-21 to IL-2 T-cell cultures seems to antagonize IL-2 induced exhaustion (Supplementary Figure 3A). Furthermore, CLL T-cells are characterized by an increased expression of PD-1 as compared to healthy donor T-cells [29], which can be reduced *in vitro* by IL-21 application (Figure 3D).

### Increased central-memory-like T-cell frequency after expansion with IL-21

**Figure 4:**
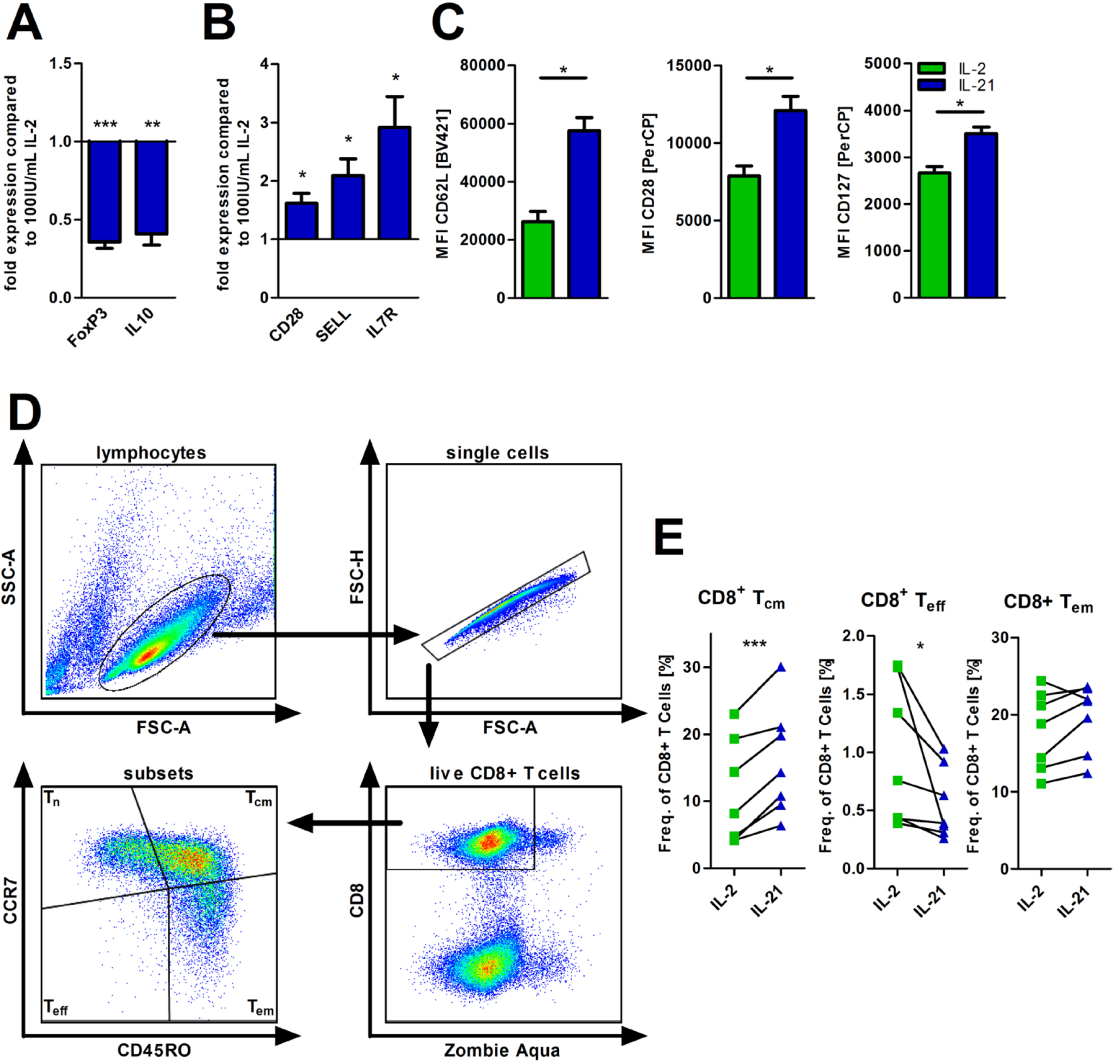
IL-21 modulates T-cell differentiation (**A**–**E**) T-cells were cultured for 5 days in the presence of IL-2 or IL-21 with anti-CD2/CD3/CD28 activation/expansion beads. (A, B) Subsequently, cells were harvested, lysed and RNA was isolated for qPCR analysis of certain differentiation markers of T-cells (*n* = 6), i.e. FoxP3 and IL10 as representative for regulatory T-cells (A) and Bcl6, SELL and CD28 as markers for naïve/memory T-cells (B). (C) Also, the surface expression of the same markers (CD28, CD62L and CD127) were semi-quantified by flow cytometry (*n* = 6). (D–E) Frequencies of several CD8+ T-cell subsets were determined via flow cytometry (*n* = 7). CD8+ T-cell subsets were defined as: T_n_ (CCR7^pos^ CD45RO^neg^), T_eff_ (CCR7^neg^ CD45RO^neg^), T_em_ (CCR7^neg^ CD45RO^pos^) and T_cm_ (CCR7^pos^ CD45RO^pos^). MFI is defined as median fluorescence intensity. Error bars indicate the standard error mean. *P-*value: ^*^P < .05; ^**^P < .01; ^***^P < .001

Enhanced FAO and an advanced antioxidative capacity are metabolic features that have been both linked to regulatory and memory T-cell phenotypes [17, 30]. Thus, we were interested, whether IL-21 promotes a skewing towards one of the two T-cell subsets. IL-21 (as compared to IL-2) was associated with reduced FoxP3 and IL-10 mRNA levels (Figure 4A) that are indicative for naturally occurring (-like) and induced Tregs [31]. In contrast, gene expression of typical naïve/memory T-cell genes such as CD28, SELL, and IL7R were increased in T-cells treated with IL-21 for 5 days (Figure 4B). In line with this data, we observed that CD28, CD62L, and CD127 expression was higher on T-cells treated with IL-21 as opposed to cells treated with IL-2 (Figure 4C). Consequently, those phenotypic changes were linked to an expansion of central memory-like T-cells (T_cm_) and a simultaneous contraction of the effector T-cell population (T_eff_) (Figure 4D–4E). The effector memory-like T-cell fraction (T_em_) remained unaffected. In fact, addition of IL-21 to IL-2 containing T-cell cultures prevents a substantial differentiation into effector and effector-memory-like T-cells with central-memory-like T-cells being slightly elevated further suggesting potential antagonizing effects of both cytokines (Supplementary Figure 3B). Removal of IL-21 and culturing the previously IL-21 treated T-cells for two more days led to reduced levels of the T_cm_ subset paralleled by an increase of T_eff_ and T_em_ cells (Supplementary Figure 3C), which suggests an (at least partial) reversibility of the IL-21 mediated effects. In fact, no negative impact on T-cell viability was observed during this time period (Supplementary Figure 3D).

### FAO-inhibition diminishes IL-21 mediated effects on T-cell differentiation

**Figure 5:**
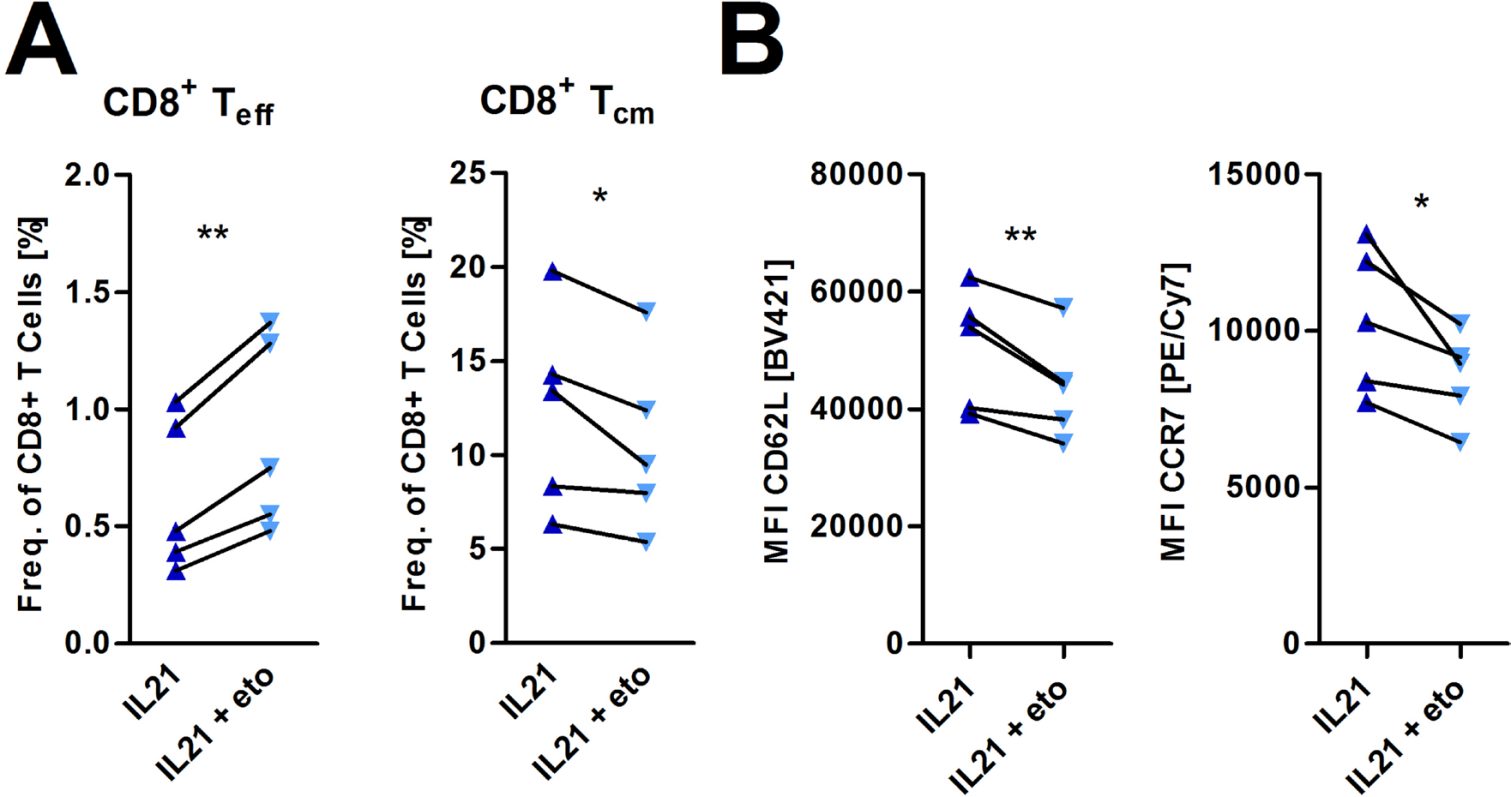
Incubation with etomoxir inhibits IL-21 mediated effects on memory T-cell differentiation (**A**, **B**) T-cells were cultured for 5 days in the presence of IL-21 and anti-CD2/CD3/CD28 activation/expansion beads with or without 40µM etomoxir. (A) Frequencies of several CD8+ T-cell subsets were assessed via flow cytometry as outlined in Figure 4D (*n* = 5). (B) Surface expression of CD62L and CCR7 was analyzed upon etomoxir treatment (*n* = 5). MFI is defined as median fluorescence intensity. *P-*value: ^*^P < .05; ^**^P < .01; ^***^P < .001

To date, increasing evidence suggests that metabolic pathways control various aspects of T-cell biology [15]. As described in previous sections, we observed an IL-21 mediated shift towards FAO (Figure 1F, 1H). FAO is described to be connected to formation of memory T-cells, which display a diminished senescence/exhaustive phenotype [17, 32]. Here, we aimed to explore whether this FAO skewing is critical for mediating immune phenotypical changes under IL-21 treatment, in particular the promotion of central-memory T-cells and antagonization of T-cell senescence/exhaustion. To do so, we used Etomoxir, an irreversible CPT1a inhibitor, in non-cytotoxic dosages (Supplementary Figure 4A). In fact, blocking FAO in IL-21 treated activated T-cells reduced the promotion of the central memory-like Tcells and the decrease of effector T-cells respectively (Figure 5A). In accordance to the attenuated differentiation into central memory-like T-cells, surface expression of CD62L and CCR7 were also reduced (Figure 5B). In terms of the senescence/exhaustion markers no changes were observed during FAO inhibition (Supplementary Figure 4B).

## DISCUSSION

To date, common gamma chain cytokines, e.g. IL-2 and IL-15, have been thoroughly studied in terms of their immunomodulatory and immune cell promoting effects. Thereby, a steadily increasing number of studies highlights how T-cell metabolism, modulated by cytokines [33], governs T-cell function with almost nothing reported about IL-21 in this context so far [34]. Here, we sought out to investigate whether IL-21 has any effects on human T-cell metabolism, which thereby influences differentiation and/or senescence.

We observed decreased aerobic glycolysis upon IL-21 treatment. In this context, a fast switch from OXPHOS in quiescent cells towards aerobic glycolysis is prototypical for activated effector T-cells but at the same time drives them towards a terminally differentiated state [35, 36]. In contrast, pharmacological inhibition of glycolysis can preserve the formation of long-lived memory T-cells with an enhanced anti-tumor activity as shown in preclinical models [37]. A mitigated glycolytic activity by IL-21 could be documented at different levels e.g. less glucose uptake, reduced expression of key glycolytic genes such as LDHA and PDK1, and the cells’ weakened dynamic glycolytic capacity. Altogether, these alterations suggest effects on central metabolic regulators such as the AMP-activated protein kinase [38], which obviously needs to be explored further.

On the other hand, IL-21 yielded (as compared to IL-2 and similar to IL-15) higher levels of CPT1a gene expression and of mitochondrial biogenesis [17]. Both, CPT1a expression and increased mitochondrial mass, contribute to an enhanced SRC that refers to an extra mitochondrial ability to produce energy under conditions of increased demand, which is typical for memory T-cells [17]. Overall, energetic substrate dependency shifted towards fatty acids. Counterintuitively, fatty acid uptake was found reduced, which could be explained by recent findings that IL-15 induced memory T-cells met their demand for fatty acids through intrinsic lipolysis [30]. Since glucose uptake was reduced, we hypothesize that glutamine (in addition to stored lipids) might fuel the *de novo* lipogenesis. In that case, glutamine is converted into glutamate by glutaminase (GLS) and shuttled into the mitochondrial TCA cycle. The TCA cycle-derived citrate is then exported into the cytosol for fatty acid synthesis [15]. However, both expression of glutamine transporters and GLS were not upregulated while dependency of mitochondrial OXPHOS on glutamine remained unchanged upon IL-21 treatment (data not shown, Supplementary Figure 1F).

Moreover, IL-15 generated memory T-cells produce less toxic superoxide radicals and have an increased mitochondrial membrane potential, which in turn contributes to their superior long-term survival [17]. It has also been shown that IL-15 exerts T-cell-protective effects by promoting thiol synthesis [12]. IL-21 elicits similar positive effects on the T-cells’ mitochondrial fitness. Furthermore, mitochondrial ROS production can also act as a second messenger during T-cell activation [39]. Therefore reducing ROS levels could be a mechanism for mitigating T-cell activation and for simultaneously promoting memory formation, which is indeed seen in IL-21 treated T-cells. In addition to the reduced mitochondrial ROS production, we observed an upregulation of antioxidants including key molecules for glutathione synthesis, hydrogen peroxide-metabolizing catalase, and the pleiotropic HO-1. Malignant diseases such as chronic lymphocytic leukemia (CLL) are characterized by abundant ROS production that can be detrimental for T-cells [4]. Treating CLL patient-derived T-cells with IL-21 similarly improved their redox status. This is of special importance since it shows efficacy in cells that have already been challenged by the highly oxidative tumor environment and also in view of therapeutic approaches using *ex vivo* expanded autologous lymphocytes. Previous studies have indicated that promoting the pool of intracellular glutathione as the main thiol-containing molecule and the expression of catalase can be beneficial for adoptive T-cell based approaches [13, 14]. In addition, HO-1-holds several immunometabolic functions beyond its antioxidative properties. It can drive mitochondrial biogenesis via TFAM induction [4, 40], which is actually found at increased levels in memory T-cells and could at least partly explain our observations. Furthermore, HO-1 mediated production of carbon monoxide can suppress T-cell activation (also in an autocrine fashion) [41–43].

Containing activation intensity is discussed as a mechanism in the progressive T-cell differentiation model for memory T-cell generation [44]. Early activation markers (CD69 and CD137) and proliferative capacity were not impacted by IL-21. However, we detected a decreased IL-2R alpha chain (CD25) expression, which is consistent with previous findings [45]. T-cell senescence and exhaustion (linked to poor function and sustained expression of inhibitory receptors) can both pose substantial obstacles during *in vitro* expansion and in cancer patients [46]. Senescence (CD28 and CD57) and exhaustion (PD-1) marker [32] changes suggested a beneficial effect of IL-21. In fact, IL-21 reduced PD-1 levels also on CLL T-cells that express PD-1 at high levels and display a so-called “pseudo-“exhaustion [27].

Since Tregs are similar to memory T-cells dependent on FAO for meeting their energetic demands we sought out to investigate, whether IL-21 promotes their expansion. Gene expression of FOXP3 and IL-10 both linked to Treg differentiation was reduced which is in line with previous observations of IL-21 counteracting Treg induction and suppressivity [21, 22]. Additionally, we observed a decreased expression of CD25 which was recently described as a possible mechanism of IL-21 mediated Treg inhibition [47]. Overall, all tested immunometabolic parameters point towards promotion of memory T-cells by IL-21, which we confirmed by the according phenotypical analyses [48]. As anticipated, preclinical data suggests similar *in vivo* effects as IL-21 application has been shown to support CD4+ and CD8+ memory Tcell formation during amongst others viral infections [19, 20, 49]. In fact, recombinant IL-21 has been utilized in early clinical trials in cancer patients with e.g. melanoma or colorectal cancer [50, 51] and observations indicate an enhanced T-cell activity. However, in depth immune compartment analyses (including T-cell differentiation and/or exhaustion) have not been performed, which might be of high interest for future trials. As previously described by Kastirr *et al.*, increase was restricted to the central memory T-cells while effector memory subsets decreased [52].

FAO has emerged as a metabolic pathway critical for memory T-cell formation [30, 38]. Interfering with FAO by CPT1a inhibition during IL-21 treatment led to a reduced frequency of memory T-cells, further corroborating the notion that various stimuli (e.g. IL-15 and IL-21) merge into one common metabolic (signaling) pathway. Furthermore, senescent/exhausted T-cells display a ROS^high^ glycolysis^high^ phenotype [53]. Here, IL-21 counteracts both. However, we did not find an interconnection between FAO and expression of senescence/exhaustion markers which still needs to be elucidated further.

Taken together, we show that IL-21 triggers an immunometabolic axis in human Tcells that comprises increased mitochondrial biogenesis and SRC, a bioenergetic switch towards FAO, improved mitochondrial fitness and cellular redox status together with a FAO-dependent enhanced formation of central memory T-cells. However, IL-21 is also described to have several other effects on T-cell differentiation *in vivo*, e.g. driving Th17 differentiation and promoting both Th1 and Th2 responses [54–57]. Consequently, Tian *et al.* proposed that IL-21 affects T-cells in a more context-specific manner [34] and our *in vitro* system might not reflect ideally the *in vivo* situation. Nevertheless, we propose that IL-21 represents a promising cytokine for expanding T-cells in view of cell-based therapies and for being used as an immunotherapeutic agent that amongst other effects promotes T-cells’ fitness.

## MATERIALS AND METHODS

### Patient samples and T-cell isolation

Samples were collected upon approval by the local ethics committee (approval numbers: 3779 (HD), 3779/289_16B (CLL)) and patients’ written informed consent. Peripheral blood mononuclear cells from CLL patients and healthy donors (HD) were obtained using Ficoll-Paque (GE Healthcare, Piscataway Township, NJ) from fresh samples. T-cells from healthy donors were isolated by magnetic bead-based negative selection (Pan T-cell isolation kit, human, Miltenyi Biotec, Bergisch Gladbach, Germany). Purity levels were routinely assessed by flow cytometry. Thereby, T-cells of at least 95% purity were used for cell culture experiments. Whole PBMCs were used for cell culture experiments with CLL patients’ samples.

### Cell culture

T-cells were cultured in complete RPMI 1640 medium supplemented with 40 U/mL Penicillin and 40 µg/mL Streptomycin (Life Technologies, Carlsbad, CA, USA), 20 mM GlutaMAX™ and 10% fetal bovine serum (FCS, PAN-Biotech, Aidenbach, Germany). CLL PBMCs with less than 70% CD5^+^ B-cells were cultured in AIM V medium (Life technologies). Cytokines and T-cell activation beads were purchased from Miltenyi Biotec. Cells were analyzed after cell culture for 24 hours or 5 days with 100 IU/mL IL-2 or 10–50 ng/mL IL-21 in the presence or absence of anti-CD2/CD3/CD28 activation beads (T-cell Activation/Expansion Kit, human, Miltenyi Biotec, Bergisch Gladbach, Germany).

### Antibodies and flow cytometry

Cells were stained using fluorochrome-coupled antibodies according to the manufacturers’ recommendations (Supplementary Table 1). Antibody staining was performed after blocking with mouse IgG (Dianova, Hamburg, Germany) and Human TruStain FcX™ (Biolegend, San Diego, California, USA). Dead cells were discriminated using the Zombie Aqua™ Fixable Viability Kit (Biolegend). For proliferation assays cells were labeled with Violet Proliferation Dye 450 (VPD450, BD Bioscience, Franklin Lakes, New Jersey, USA) before cell culture. Cells were analyzed on a FACS Canto II flow cytometer (BD Bioscience). Subsequent evaluations were done with the FlowJo Version 10.1 (TreeStar, Ashland, OR, USA).

### Detection of intracellular oxidative stress and glutathione

Total intracellular levels of ROS were detected by staining with CellROX™ Deep Red Reagent. Mitochondria-specific superoxide production was assessed using MitoSOX™ Red. For detection of intracellular glutathione cells were stained with ThiolTracker™ Violet. After the staining fluorescence intensities were semiquantified by flow cytometry. All three probes were purchased from Thermo Fisher Scientific.

### Mitochondrial membrane potential

Differences of mitochondrial membrane potential were assessed using the potentiometric dye JC-1 (JC-1 Mitochondrial Assay Kit, Cayman Chemical). Therefore, cells were stained with JC-1 according to the manufacturer’s instructions for 15 minutes at 37° C followed by flow cytometry analysis.

### Glucose and fatty acid uptake assays

Influx of glucose was semi-quantified by flow cytometry based on the uptake of the fluorescent glucose analogue 6-(N-(7-Nitrobenz-2-oxa-1,3-diazol-4-yl)amino)-2-deoxyglucose (6-NBDG, Thermo Fisher Scientific) into the cells using the staining protocol recommended by the manufacturer. For assessing uptake of long-chain fatty acids, T-cells were harvested, washed with RPMI without FCS and incubated with the green fluorescent Bodipy Fl C_16_ (Thermo Fisher Scientific) at 37° C. Afterwards, cells were washed with ice-cold PBS + 10% FCS and subsequently semiquantified by flow cytometry.

### DNA/RNA preparation and quantitative polymerase chain reaction (qPCR)

RNA and DNA were extracted from cell lysates with either the innuPREP RNA Mini Kit for assessing gene expression or the innuPREP DNA/RNA Mini Kit (both Analytik Jena AG) for determining mitochondrial DNA (mtDNA) content. cDNA was synthesized using SuperScript II Reverse Transcriptase system (Thermo Fisher Scientific) in combination with a Mastercycler Nexus (Eppendorf, Hamburg, Germany). mRNA levels were quantified with QuantiTect SYBR Green PCR Kit on a Rotor Gene Q (Qiagen, Hilden, Netherlands). Relative gene expression was afterwards calculated by normalizing the expression of each target gene to β-actin using gene-specific primers (Supplementary Table 2). For determining mtDNA content, ratios were calculated with c_t_-values of tRNA ^Leu(UUC)^ normalized to β2microtubulin. Afterwards relative mtDNA content was determined with ratios of IL-2 set as 100%.

### Fluorescence microscopy of mitochondria

Cells were stained with MitoTracker® Green FM for mitochondria visualization (Life technologies) and DAPI (Sigma Aldrich, St. Louis, MO). Afterwards, cells were immediately analyzed using a fluorescence microscope (LSM710, Zeiss, Jena, Germany). Fluorescence intensity was assessed with ImageJ (NIH, Bethesda, Maryland, USA) for five single cells of each condition and donor.

### Glucose and lactic acid measurements

Glucose and lactic acid concentrations of cell culture supernatants were determined using Super GL Compact (Hitado, Möhnsee, Germany).

### Extracellular flux assays

Measurements were performed using a Seahorse XFe96 flux analyzer and the corresponding kits (Agilent, Santa Clara, California, USA). Glycolysis/Mitochondrial Stress Test Assays were performed as detailed before [4]. Dependencies on glucose, glutamine, and fatty acid pathways were studied using the Mito Fuel Flex Test Assay as recommended by the manufacturer. Within this assay, three inhibitors were used: UK5099 (glucose oxidation pathway), BPTES (glutaminase), and Etomoxir (FAO). For testing bioenergetic dependency on exogenous fatty acids, we added BSA-conjugated palmitate into the wells (with T-cells) right before the assay and measured oxygen consumption rate at baseline conditions (= basal rate) and after injection of 200 µM Etomoxir and/or of 200 µM SSO (Cayman Chemical/Biomol, Hamburg, Germany).

### Western blot

Protein lysates were prepared from T-cell pellets using RIPA (Sigma-Aldrich) solution and the Halt™ Protease/Phosphatase Inhibitor Single-Use Cocktail (Thermo Fisher Scientific). Protein concentrations were determined using a BCA protein assay Kit (Thermo Fisher Scientific). Proteins (>10 µg) were separated on a sodium dodecyl sulphate-polyacrylamide gel electrophoresis (SDS-PAGE) system and transferred by wet blotting (Biorad, Hercules, California, USA). Western blots were probed using primary antibodies against Tubulin, CPT1a, Bcl-2, Bcl-xL, and Mcl-1 (Cell Signaling Technology Inc., Danvers, Massachusetts, USA and Supplementary Table 3). Bands were detected using horseradish peroxidase-conjugated anti-rabbit or anti-mouse IgGs (Cell Signaling Technology; Agilent) and an ECL substrate (Cell Signaling Technology Inc.).

### IL-21 enzyme-linked immunosorbent assay

Detection of IL-21 in patients’ and healthy donor serum samples was performed by sandwich ELISA using a monoclonal capture and a monoclonal biotin-conjugated detection antibody from Biolegend. The detection limit of this assay is at 16 pg/mL.

### Statistical analysis

Differences in means and medians were evaluated with parametric or nonparametric methods based on the distribution levels. All statistical analyses were performed using GraphPad Prism Version 5 (GraphPad Prism Software Inc., La Jolla, California, USA).

## SUPPLEMENTARY MATERIALS FIGURES AND TABLES



## Abbreviations

IL-2: Interleukin-2
IL-21: Interleukin-21
ECAR: extracellular acidification rate
Eto: etomoxir
FAO: fatty acids oxidation
OCR: oxygen consumption rate
OXPHOS: oxidative phosphorylation
ROS: reactive oxygen species
SRC: spare respiratory capacity
Treg: regulatory T-cell
T_cm_: central memory T-cell
T_eff_: effector T-cell
T_em_: effector memory-like T-cell
