# Supplementary material for: IL-21 modulates memory and exhaustion phenotype of T-cells in a fatty acid oxidation-dependent manner
Source: Oncotarget. 2018 Feb 7;9(17):13125–38. doi: 10.18632/oncotarget.24442 (PMC5862566; doi:10.18632/oncotarget.24442)
Supplement: Supplementary file 1 [file oncotarget-09-13125-s001.pdf]

IL-21 modulates memory and exhaustion phenotype of T-cells in a fatty acid oxidation-dependent manner

SUPPLEMENTARY MATERIALS

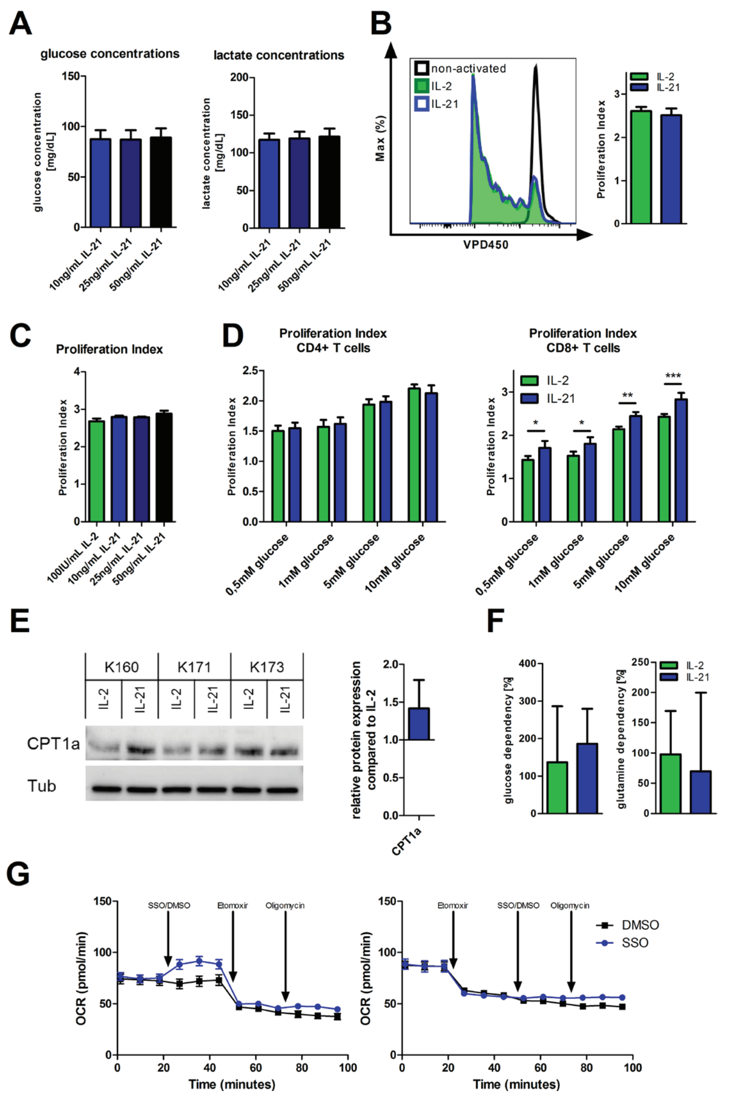

Supplementary Figure 1: Metabolic dependencies of IL-21 treated T-cells. (A–G) T-cells were cultured for 5 days in the presence of IL-2 or IL-21 with anti-CD2/CD3/CD28 activation/expansion beads. (A) Supernatants from T-cell cultures treated with different IL-21 concentrations were collected (n = 6) and glucose/lactate content determined using a HITADO Super GL compact device. (B) Initially, T-cells were stained with VPD450 and cultured for 5 days. Afterwards, proliferation was assessed by flow cytometry (n = 6). (C) The impact of increasing IL-21 concentrations on T-cell proliferation was determined by flow cytometry (n = 3). (D) Proliferation of $CD4^+$ and $CD8^+$ T-cells cultured under different glucose concentrations in presence of IL-2 or IL-21 was assessed by flow cytometry (n = 5). (E) Protein levels of CPT1a were detected by Western Blot and semiquantified by ImageJ software (n = 3). Band intensities were normalized to Tubulin and the values of IL-2 treated T-cells were set as 100%. (F) Dependency on glucose and glutamine for fueling OXPHOS was tested using the Seahorse Mito Fuel Flex tests (n = 3). For glucose dependency, blocking of the mitochondrial pyruvate carrier with UK5099 led to a shutdown of glucose fueling to the TCA. Conversion of glutamine to glutamate via glutaminase was inhibited with BPTES. Dependency is defined as the difference between baseline respiration and oxygen consumption after inhibition of one metabolic pathway. Error bars indicate the standard deviation. (G) Influence of exogenous fatty acids on OXPHOS was tested by adding fatty acids before the start of the flux analysis and the consecutive injection of 200 µM SSO, a CD36 inhibitor, and/or 200 µM etomoxir, a CPT1a inhibitor (n = 3). Unless otherwise stated, error bars indicate standard error means. P-value: $^*P < .05$.

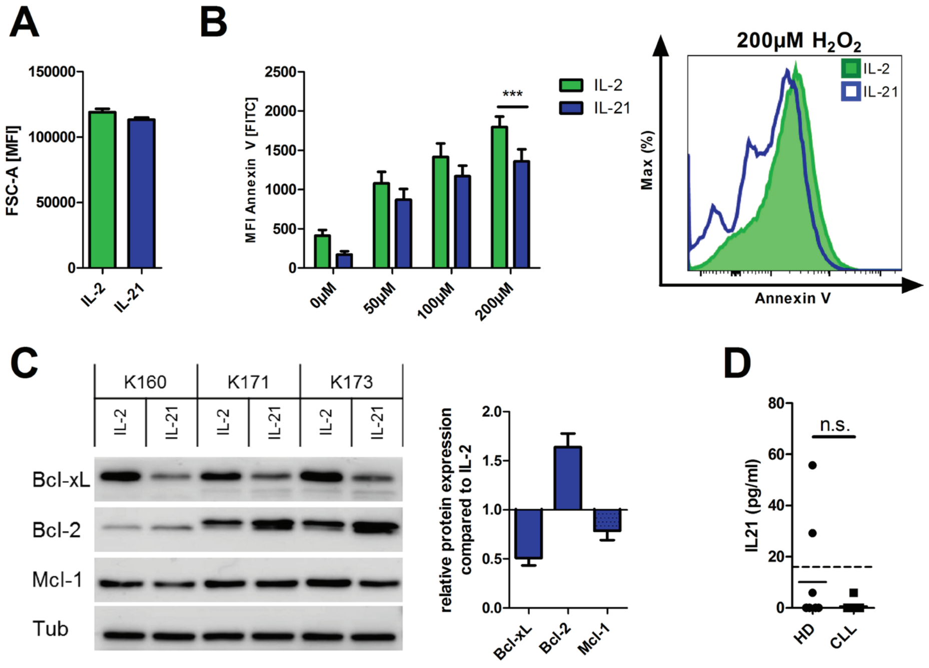

Supplementary Figure 2: Expansion with IL-21 promotes expression of Mcl-1 in T-cells and their resilience towards ROS. (**A**) Comparison of the T-cells' size using the forward scatter index as assessed by flow cytometry (n = 6). (**B**) After expansion for 5 days, T-cells were exposed to 200 µM of H_2O_2 for 24 h and stained for Annexin V. (Left panel) MFIs of 5 different donors are displayed. (Right panel) Histogram shows a flow-cytometry-based comparison of the Annexin V staining intensity between IL-2 and IL-21 treated T-cells of one representative donor. (**C**) Protein expression of Bcl-2, BclxL, and Mcl-1 was analyzed by Western Blot. Band intensities were measured with ImageJ and normalized to Tubulin. Values obtained for IL-2 treated Tcells were set as 100%. (**D**) IL-21 concentrations in healthy donor-(n = 10) and CLL patient-derived sera (n = 10) were analyzed using an IL-21 ELISA. The dashed line indicates the detection limit of this assay. MFI is defined as median fluorescence intensity. Error bars indicated the standard error means.

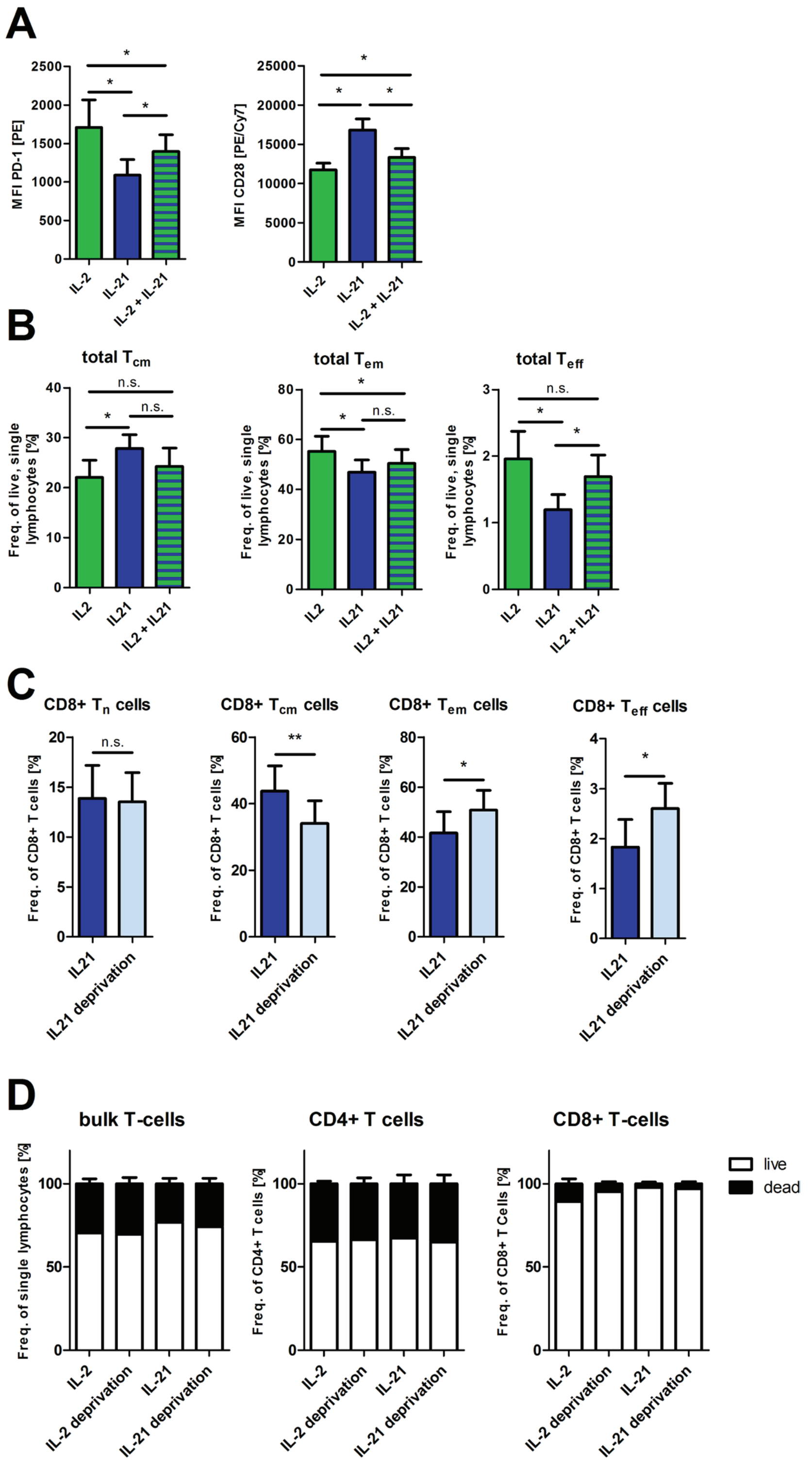

Supplementary Figure 3: IL-21 attenuates the IL-2 mediated effects on T-cell exhaustion and differentiation. (**A**, **B**) T-cells were cultured for 5 days in the presence of 100 IU/mL IL-2 and/or 10 ng/mL IL-21. (A) Surface expression of PD-1 and CD28 was assessed by flow cytometry (n = 6). (B) Frequencies of central memory-like, effector memory-like, and effector T-cells were determined by flow cytometry. Here, frequencies of total T-cells are depicted (n = 5). (**C**, **D**) T-cells were harvested after expansion for 5 days und cultured for additional two days in presence or absence of cytokines as indicated (n = 6). (C) Frequencies of central memory-like, effector memory-like, and effector T-cells were determined by flow cytometry. (D) Cells were stained with Annexin V/7AAD to determine live and dead cells. Dead cells are defined as Annexin V$^+$ and/or 7AAD-positive$^+$ cells. MFI is defined as median fluorescence intensity. Error bars indicate standard error means.

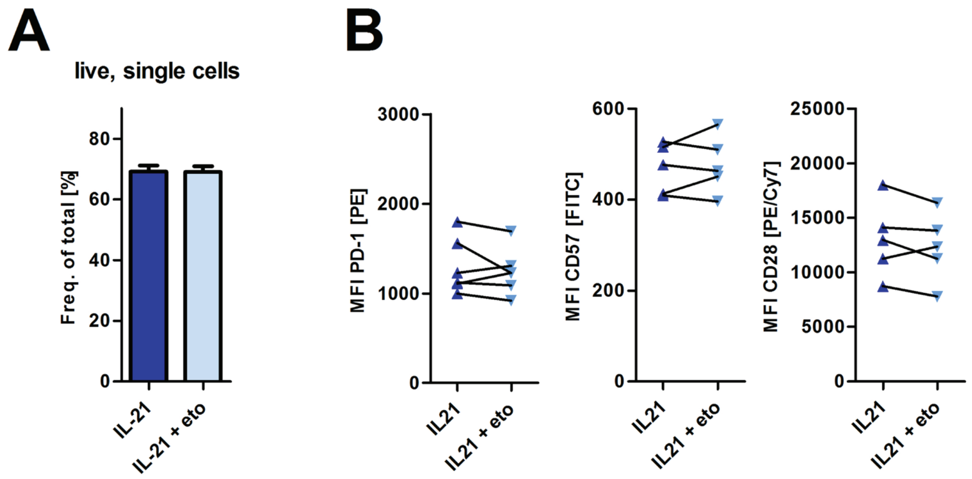

Supplementary Figure 4: Influence of etomoxir on T-cell viability and expression of senescence/exhaustion markers. (**A**, **B**) T-cells were cultured for 5 days in the presence of IL-21 and anti-CD2/CD3/CD28 activation/expansion beads with or without 40 µM of the CPT1a inhibitor etomoxir. (A) Assessment of viability was accomplished using the Zombie Aqua dye ($n = 6$). (B) Surface expression of CD28, PD-1, and CD57 upon CPT1a inhibition was assessed by flow cytometry ($n = 5$). MFI is defined as median fluorescence intensity. Error bars indicate standard error means.

Supplementary Table 1: Fluorochrome-coupled antibodies used for flow cytometry

Antibody	Fluorochrome	Clone	Isotype	Company
CD3	APC	UCHT1	Mouse IgG1, κ	Biolegend
CD3	Pacific Blue	UCHT1	Mouse IgG1, κ	Biolegend
CD4	FITC	OKT4	Mouse IgG2b, κ	Biolegend
CD4	APC/Cy7	OKT4	Mouse IgG2b, κ	Biolegend
CD8a	FITC	HIT8a	Mouse IgG1, κ	Biolegend
CD8a	APC	RPA-T8	Mouse IgG1, κ	Biolegend
CD25	APC	M-A251	Mouse IgG1, κ	Biolegend
CD28	PE/Cy7	CD28.2	Mouse IgG1, κ	Biolegend
CD28	PerCP/Cy5.5	CD28.2	Mouse IgG1, κ	Biolegend
CD36/FAT	FITC	5–271	Mouse IgG2a,κ	Miltenyi
CD45RO	PE	UCHL1	Mouse IgG2a, κ	Biolegend
CD57	FITC	HCD57	Mouse IgM, κ	Biolegend
CD62L	Brilliant Violet 421	DREG-56	Mouse IgG1, κ	Biolegend
CD69	PerCP/Cy5.5	FN50	Mouse IgG1, κ	Biolegend
CD127	PerCP Cy5.5	A019D5	Mouse IgG1, κ	Biolegend
CD137	PE/Cy7	4B4–1	Mouse IgG1, κ	Biolegend
CD197 (CCR7)	Alexa Fluor 647	G043H7	Mouse IgG2a, κ	Biolegend
CD197 (CCR7)	PE/Cy7	G043H7	Mouse IgG2a, κ	Biolegend
CD279 (PD-1)	PE	EH12.2H7	Mouse IgG1, κ	Biolegend
GLUT1	APC	202915	Mouse IgG2B	R&D systems

Supplementary Table 2: Primers used for qPCR analysis

Gene	Primer
actin	QT01680476/Qiagen
cat	QT00079674/Qiagen
cd28	QT00001267/Qiagen
cpt1a	QT00082236/Qiagen
foxp3	QT00048286/Qiagen
gclc	QT00037310/Qiagen
gclm	QT00038710/Qiagen
hmox1	QT00092645/Qiagen
il-7r	QT00053634/Qiagen
il10	QT00041685/Qiagen
ldha	QT00001687/Qiagen
pdk1	QT00069636/Qiagen
sell	QT00014497/Qiagen
for determining mtDNA content	
beta2microtubulin	beta2M F594: TGCTGTCTCCATGTTTGATGTATCT
	beta2M R679: TCTCTGCTCCCCACCTCTAAGT
mtDNA tRNA $^{Leu(UUC)}$	tRNA F3212: CACCCAAGAACAGGGTTTGT
	tRNA R3319: TGGCCATGGGTATGTTGTTA

Supplementary Table 3: Antibodies used for Western Blot

Antibody	Species/Isotype	Company
**primary**		
Bcl-2	rabbit IgG	Cell Signaling Technology
Bcl-xL	rabbit IgG	Cell Signaling Technology
CPT1a	rabbit IgG	Cell Signaling Technology
Mcl-1	rabbit IgG	Cell Signaling Technology
Tubulin	mouse IgG1, κ	Abcam
**secondary**		
anti-mouse-HRP	goat	Dako
anti-rabbit-HRP	goat	Cell Signaling Technology
